# Supplementary material for: Integrated mRNA and miRNA Transcriptome Analysis Suggests a Regulatory Network for UV–B-Controlled Terpenoid Synthesis in Fragrant Woodfern (Dryopteris fragrans)
Source: Int J Mol Sci. 2022 May 20;23(10):5708. doi: 10.3390/ijms23105708 (PMC9148142; doi:10.3390/ijms23105708)
Supplement: Supplementary file 1 [file ijms-23-05708-s001.zip › Supplementary Figure.pdf]

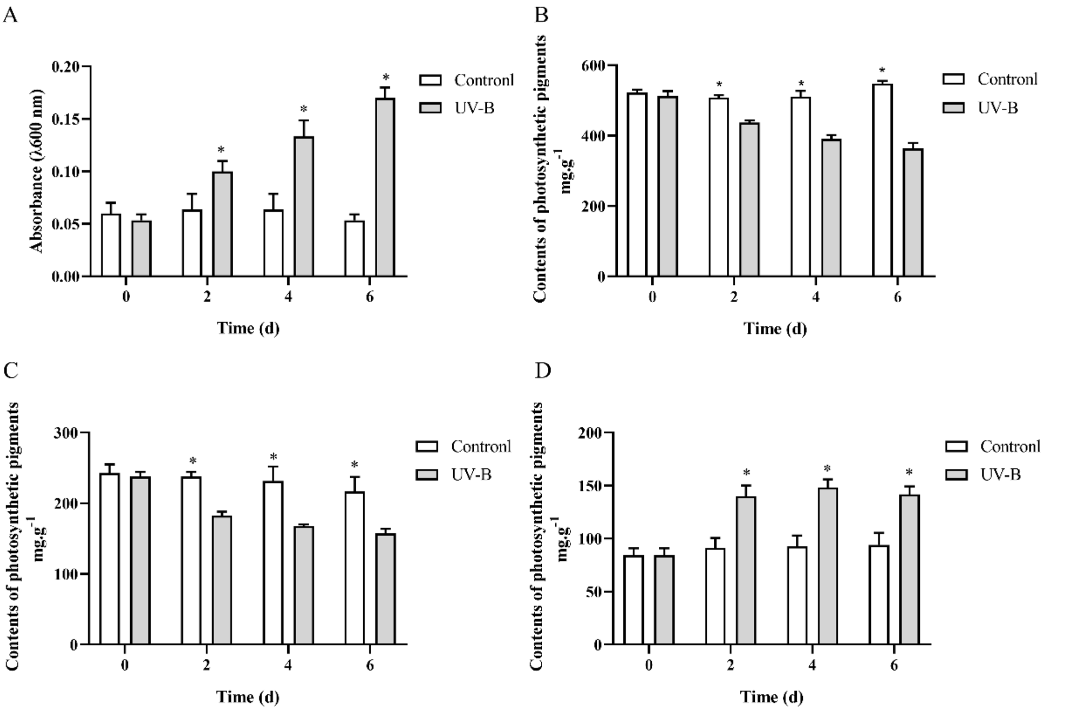

Supplementary Figure S1. Changes of cell death estimated by Evans blue staining and photosynthetic pigments in Fragrant Woodfern plants after UV-B treatment. A, Changes of cell death estimated by Evans blue staining in Fragrant Woodfern plants after UV-B treatment; B, Changes of chlorophyll a content in Fragrant Woodfern plants after UV-B treatment; C, Changes of chlorophyll b content in Fragrant Woodfern plants after UV-B treatment; D, Changes of total carotenoids content in Fragrant Woodfern plants after UV-B treatment; * $p < 0.05$

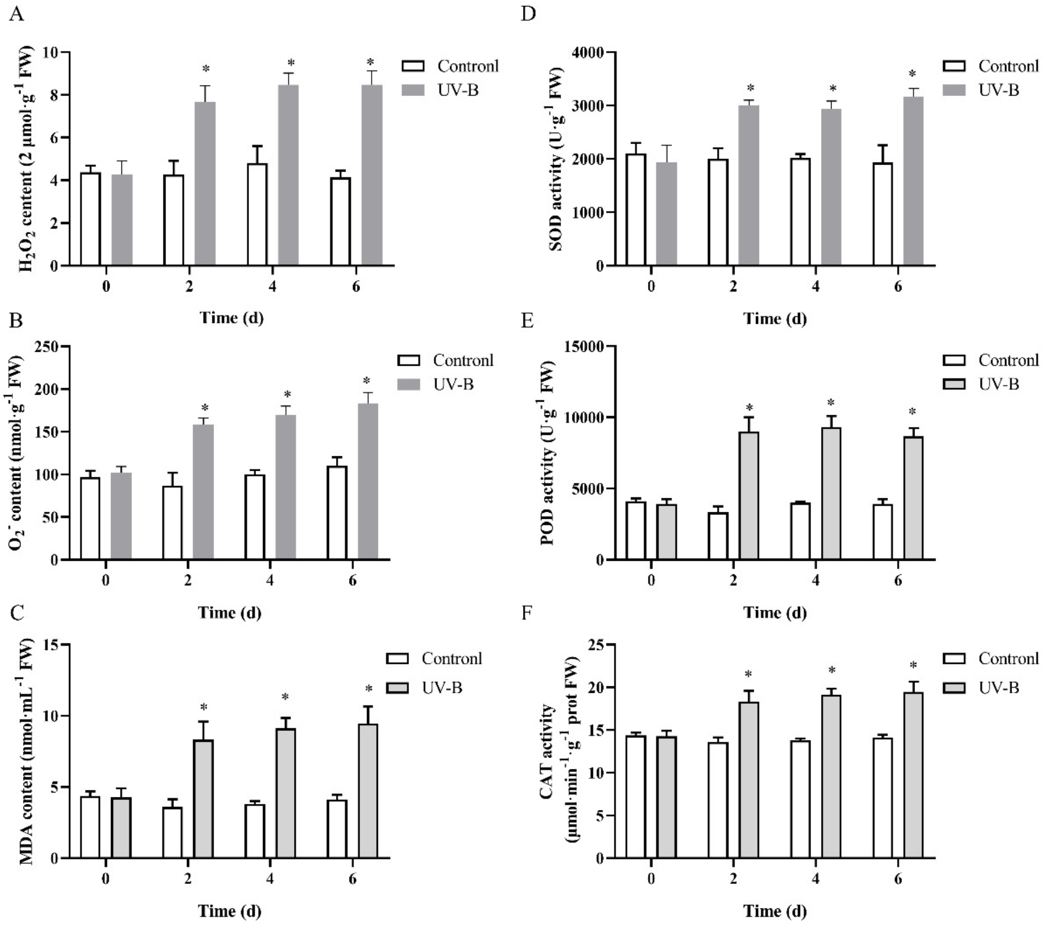

Supplementary Figure S2. Effect of different UV-B radiation time on the contents of H_2O_2 (A), O_2^- (B), and MDA (C), and the activities of SOD (D), POD (E), and CAT (F) in Fragrant Woodfern seedlings. * $p < 0.05$

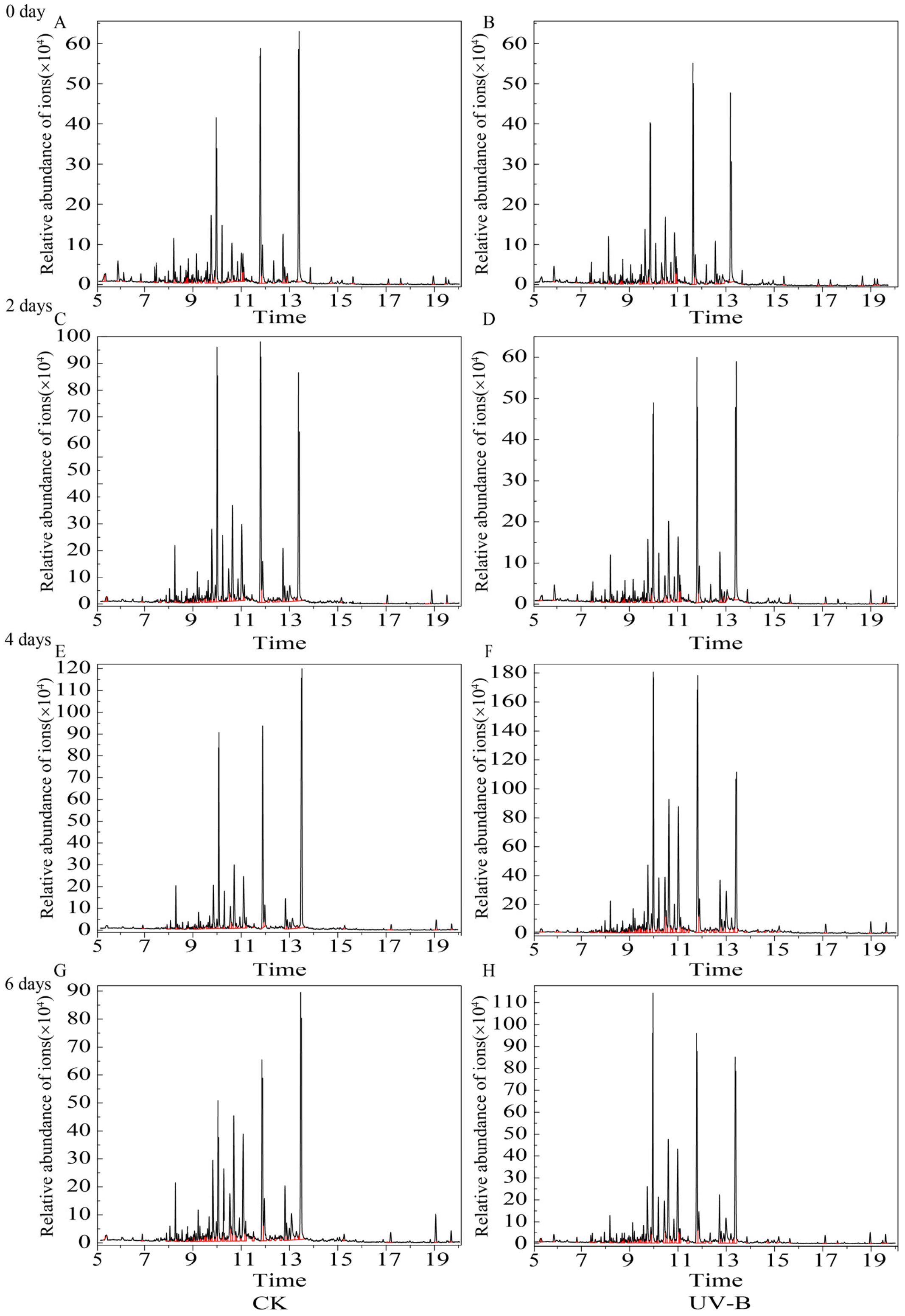

Supplementary Figure S3. Effect of different UV-B radiation time on the contents of terpenoid in Fragrant Woodfern seedlings. (A), Control group of 0 day on the contents of terpenoid in Fragrant Woodfern seedlings; (B), UV-B radiation of 0 day on the contents of terpenoid in Fragrant Woodfern seedlings; (C), Control group of 2 day on the contents of terpenoid in Fragrant Woodfern seedlings; (D), UV-B radiation of 2 day on the contents of terpenoid in Fragrant Woodfern seedlings; (E),

Control group of 4 day on the contents of terpenoid in Fragrant Woodfern seedlings; (F), UV-B radiation of 4 day on the contents of terpenoid in Fragrant Woodfern seedlings; (G), Control group of 6 day on the contents of terpenoid in Fragrant Woodfern seedlings; (H), UV-B radiation of 6 day on the contents of terpenoid in Fragrant Woodfern seedlings.

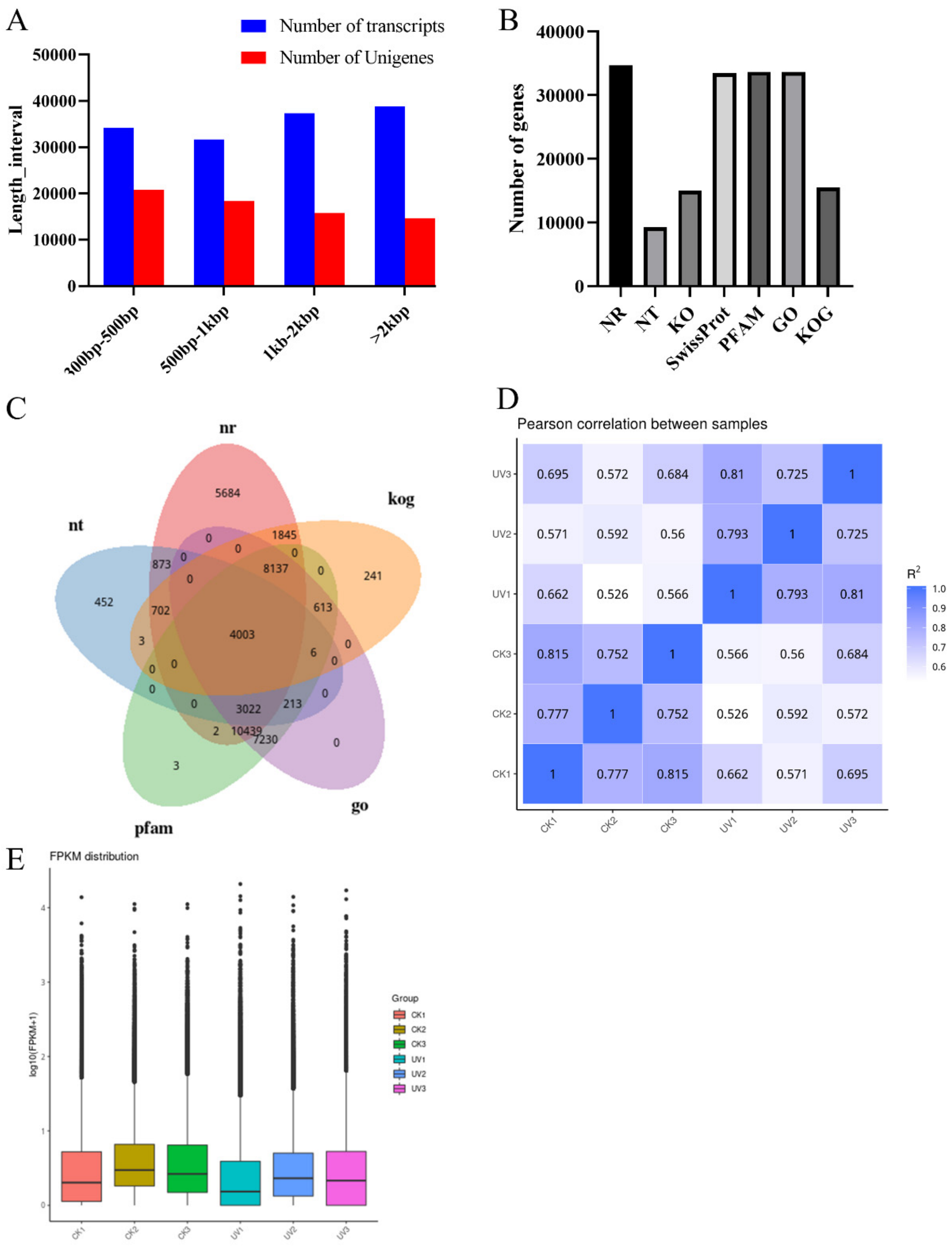

Supplementary Figure S4. The annotation of Fragrant Woodfern leaves assembled mRNA transcriptome and gene expression profiling. A, Length distribution of assembled cultivar Fragrant Woodfern mRNA transcripts and genes, the abscissa represents the length; B, Number of genes aligned to different databases; C, Venn diagram showing the numbers of shared and unique expressed genes across the 7 functional database; D, Correlation analysis between samples replicates. E, FPKM Box-plot density distribution gene expression.

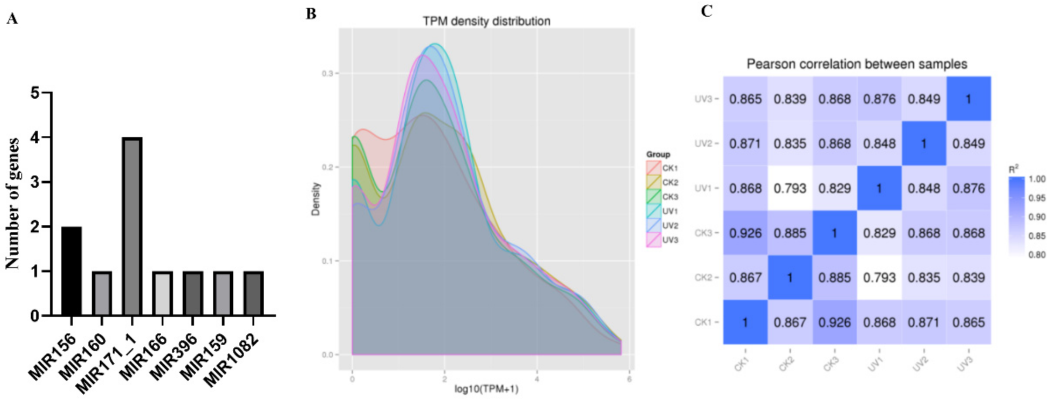

Supplementary Figure S5. The annotation of Fragrant Woodfern leaves assembled miRNA transcriptome and gene expression profiling. A, Number of genes belonging to different 10 miRNA families; B, TPM density distribution chart; C, Correlation analysis between samples replicates.

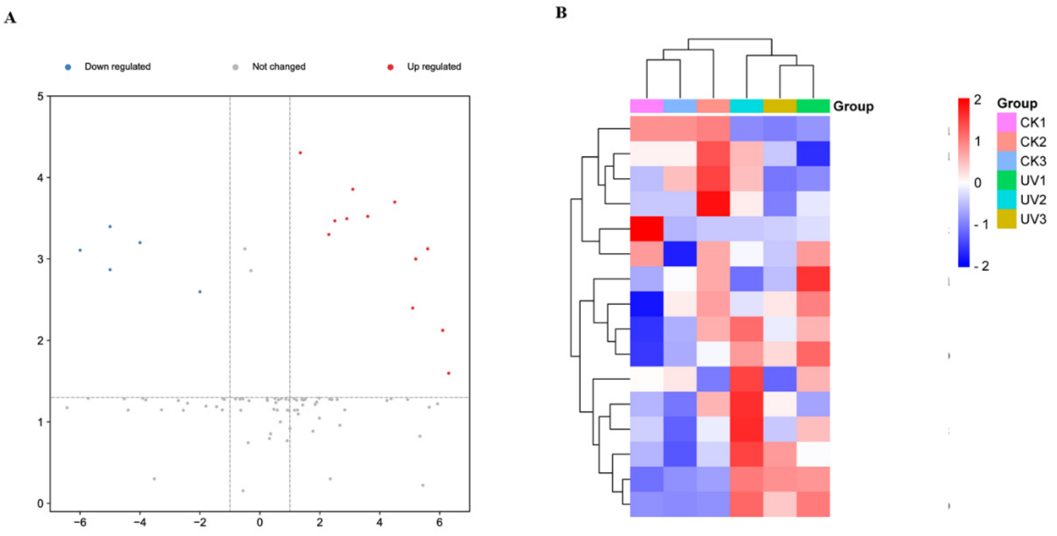

Supplementary Figure S6. Differential expression miRNAs were obtained under UV-B treatment in Fragrant Woodfern leaves. A, volcano map representing the levels of DEMs; B, Heat map representing the levels of DEMs.

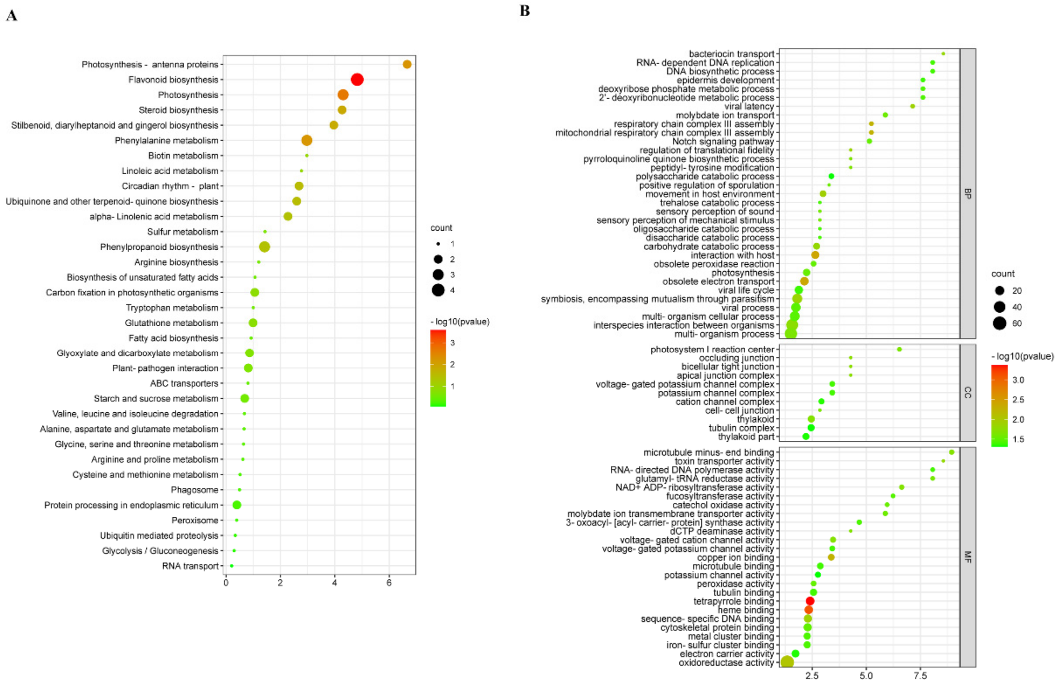

Supplementary Figure S7. GO (A) and KEGG (B) functional classification of DEMs.
