# Supplementary material for: Integrated mRNA and miRNA Transcriptome Analysis Suggests a Regulatory Network for UV–B-Controlled Terpenoid Synthesis in Fragrant Woodfern (Dryopteris fragrans)
Source: Int J Mol Sci. 2022 May 20;23(10):5708. doi: 10.3390/ijms23105708 (PMC9148142; doi:10.3390/ijms23105708)
Supplement: Supplementary file 1 [file ijms-23-05708-s001.zip › Supplementary Table .pdf]

Supplementary Table S1. GC-MS detection of contents of terpenoid in Fragrant Woodfern

	min	CAS No	description	Molecular Formula	0 CK	0 UV-B	2 CK	2 UV-B	4 CK	4 UV-B	6 CK	6 UV-B
1	5.341	31983-22-9	α-Muurolene	$C_{15}H_{24}$	2.85±0.05	5.37±1.25	1.68±1.01	3.29±0.2	0.00	1.96±0.02	0.41±0.02	3.08±1.02
2	6.848	116-04-1	β-HUMULENE	$C_{15}H_{24}$	2.07±0.07	1.46±0.9	1.18±1.02	1.35±0.02	0.93±0.02	1.85±0.08	1.33±0.12	1.45±0.07
3	7.988	544-76-3	hexadecane	$C_{16}H_{34}$	2.12±0.1	1.90±0.7	1.88±0.9	2.20±0.1	1.52±0.02	3.13±0.9	1.98±0.05	3.14±1.02
4	8.209	143-13-5	nonyl acetate	$C_{11}H_{22}O_2$	8.64±0.9	8.64±1.25	8.64±1.08	8.64±1.02	8.64±1.02	8.64±1.02	8.64±1.02	8.64±1.07
5	8.274	540-97-6	Cyclohexasiloxane,dodecamethyl	$C_{12}H_{36}O_6Si_6$	2.15±1.01	1.73±0.7	1.09±0.05	1.66±0.01	1.03±0.01	1.68±0.01	1.28±0.04	1.31±0.07
6	8.344	3891-98-3	2,6,10-trimethyldodecane	$C_{15}H_{32}$	0.00	0.00	0.89±0.05	1.01±0.07	0.85±0.01	1.35±0.02	0.95±0.07	1.33±0.04
7	8.49	6831-16-9	(-)-ARISTOLENE	$C_{15}H_{24}$	2.92±0.9	1.70±0.9	1.87±1.01	2.00±0.2	1.34±0.02	2.55±0.01	1.71±0.07	2.34±0.5
8	8.663	17334-55-3	(+)-CALARENE	$C_{15}H_{24}$	1.05±0.9	0.00±	1.10±0.02	1.87±0.05	0.57±0.02	1.30±0.03	1.01±0.01	1.54±0.04
9	8.776	1139-17-9	(-)-ISOLONGIFOLOL	$C_{15}H_{26}$	1.84±0.7	1.31±0.04	2.36±0.9	1.41±0.05	1.26±0.01	1.43±0.05	2.16±0.07	3.29±0.07
10	9.225	26620-71-3	Naphthalene,1,2,3,4,6,7,8,8a-octahydro-1,8a-dimethyl-7-(1-methylethenyl)-, (1R,7R,8aS)-	$C_{15}H_{24}$	2.35±1.02	1.80±0.07	2.51±0.09	2.27±0.04	1.64±0.01	3.72±0.9	2.49±0.09	4.11±1.02
11	11.883	19078-37-6	1,4,4a,5,6,7,8,8a-Octahydro-2,5,5,8a-tetramethyl-1-naphthalenemethanol	$C_{15}H_{26}$	13.08±2.07	8.86±1.02	7.56±1.02	12.57±1.02	6.68±1.02	21.17±2.05	12.64±1.02	17.58±2.05
12	13.39	72345-17-6	1H-Cyclopropa[a]naphthalene, 1a,2,3,3a,4,5,6,7b-octahydro-1,1,3a,7-tetramethyl-, (1aS,3aR,7bR)-	$C_{15}H_{24}$	9.25±1.08	32.90±2.05	17.46±2.5	32.29±5.04	23.83±2.05	34.75±3.05	22.28±2.07	23.12±7.07

13	17.059	192724-27-9	(-)-drimenol	$C_{15}H_{26}O$	0.00	1.78±1.02	0.00	1.98±0.2	1.78±0.05	4.16±0.9	2.84±0.07	4.26±0.9
14	18.959	523-47-7	β-Cadinene	$C_{15}H_{24}$	3.04±1.01	3.00±0.9	3.72±1.02	4.00±1.02	3.57±0.9	5.65±0.9	6.81±1.02	5.23±0.9
15	19.591	112-95-8	N-EICOSANE	$C_{20}H_{42}$	0.00	1.69±0.07	1.91±0.01	2.08±0.02	1.64±0.02	3.98±0.8	2.30±0.07	3.95±0.9
	SUM				51.35±5.7	72.13± 2.87 **	53.839±8.02	78.622±6.09 **	55.29±5.02 **	97.32±12.25 **	68.83 ±9.02 **	84.37±10.2**

$p < 0.01$ (**)

Supplementary Table S2. DEGs and DEGs primers used for qRT-PCR and gene cloning

ID	Forward primer (5'-3')	Reverse primer (5'-3')	
DfGGPS1	AAGCCACTAATCACAAGCAA	TGATGACTCGCACAACCC	qRT-PCR
DfGGPS2	ATCCATCTCCAGCAGTCAC	CAAGGGTAGCAACCACAG	qRT-PCR
DfDELLA	GGGAGATTGTGAACATAGTGG	AAATGCAAGGGCTGAAAC	qRT-PCR
DfMYC2	GCCTCTCCAATCTTAGATGGCTGCT	GTTGCAAATGATGTGGCAACCTAAC	qRT-PCR
DfSPL3	TGTGGGTTTAGTGGATGGA	GGTGAAGATGATGGAGGTGT	qRT-PCR
DfSPL6	AAGAGCCCATACCTTTGTT	GGTTGTTTCGCCTCGTTG	qRT-PCR
DfSPL9	TCCTCTTCTTGGGACTGG	AACCTCGGTGTCACTTGG	qRT-PCR
DfJAZ	AAGGACCGGGTTGAAACA	GTTGAAGGGAGTGAAGGT	qRT-PCR
Df18sRNA	GCTTTCGCAGTAGTTCGTCTTTC	TGGTCCTATTATGTTGGTCTTCGG	qRT-PCR
DfFPS1	TTTACCTTCCGGTTGCTTGT	TGAACAATCAGCCAAGAGCA	qRT-PCR
DfFPS2	TCCAGTTGCATGTGCTCTTT	ACAATCAGCCAGGAGCATTT	qRT-PCR
dfr-miRNA171a	CGAGCGAGAGCGATATTGGCT	mRQ 3' Primer	qRT-PCR
dfr-miRNA171b	TGTGTCTGTGATATTGACA	mRQ 3' Primer	qRT-PCR
dfr-miRNA171c	ACGGATCATCGAAATCGAT	mRQ 3' Primer	qRT-PCR
dfr-miRNA156b	TGGACTGCTGCTGACAGAAGA	mRQ 3' Primer	qRT-PCR
dfr-miRNA156c	GGAGACTGATTTGACAGA	mRQ 3' Primer	qRT-PCR
dfr-miRNA160a	AAAATCACTCTGCCTGGCTCCC	mRQ 3' Primer	qRT-PCR
dfr-miRNA408	ATTTAGTAGTAGCTAGGGAGAGA	mRQ 3' Primer	qRT-PCR
DfGGPS1	ATGGCGTCTGCAGCAGGGT	TTAATTCTGCCGTGAGGAGATG	Gene clone
pCAMBIA2301-DfGGPS1	GGGCATCGATACGGGATCCAT ATGGCGTCTGCAGCAGGGT	TCGAGCTCGATGGATCCCGTA TAATTCTGCCGTGAGGAGATG	Gene clone
ProDfGGPS1	GGCAACATCTTAATTGGCTTGCCAA	CTCCTATGCCAACGTCATCTCCTAT	Gene clone
pHIS2- ProDfGGPS1	ACTCACTATAGGGCG GGCAACATCTTAATTGGCTTGCCAA	CGTGAGCTCCCCGGG CTCCTATGCCAACGTCATCTCCTAT	Gene clone
DfSPL3	ATGGACAACTTGCGTCTGGA	TCACATGATAGTAGATCTGCCGA	Gene clone
pCAMBIA2301- DfSPL3	GGGCATCGATACGGGATCCAT ATGGACAACTTGCGTCTGGA	TCGAGCTCGATGGATCCCGTA TCACATGATAGTAGATCTGCCGA	Gene clone
pGADT7- DfSPL3	GGGCATCGATACGGGATCCAT ATGGACAACTTGCGTCTGGA	TCGAGCTCGATGGATCCCGTA TCACATGATAGTAGATCTGCC	Gene clone
dfr-miRNA156b	TGGACTGCTGCTGACAGAA	TCGAAAGATTGATGACAGAAG	Gene clone
pCAMBIA2301-dfr-miRNA156b	GGGCATCGATACGGGATCCAT TGGACTGCTGCTGACAGAA	TCGAGCTCGATGGATCCCGTA TCGAAAGATTGATGACAGAAG	Gene clone

Supplementary Table S3. Related genes of UV-B regulation of terpenoid synthesis in Fragrant Woodfern leaves.

ID	Fold change	EC		Description
glycolysis				
Cluster-123.15940	-1.484	4.1.2.13	ALDO	Fructose-bisphosphate aldolase, cytoplasmic isozyme
Cluster-123.16916	-1.8493		ALDO	Fructose-bisphosphate aldolase 3, chloroplastic
Cluster-123.18173	-2.621	3.1.3.11	FBP	Fructose-1,6-bisphosphatase, chloroplastic
Cluster-123.8673	2.7374	2.7.1.11	PFK	ATP-dependent 6-phosphofructokinase 6
Cluster-123.28650	3.323		PFK	ATP-dependent 6-phosphofructokinase 6
Cluster-123.30136	1.9665	4.2.1.47	GMDS	GDP-mannose 4,6 dehydratase 2
Cluster-123.5352	2.7226	1.2.1.12	GAPDH	Glyceraldehyde-3-phosphate dehydrogenase, cytosolic
Cluster-22181.1	-5.3407		GAPDH	Glyceraldehyde-3-phosphate dehydrogenase
Cluster-123.4001	5.2921	2.7.2.3	PGK	Phosphoglycerate kinase
Cluster-123.16299	1.6082	1.2.1.9	gapN	NADP-dependent glyceraldehyde-3-phosphate dehydrogenase
Cluster-123.17683	3.3096	5.4.2.11	PGAM	2,3-bisphosphoglycerate-dependent phosphoglycerate mutase
Cluster-123.21514	-1.8882	5.4.2.12	gpmI	2,3-bisphosphoglycerate-independent phosphoglycerate mutase
Citrate cycle (TCA cycle)				
Cluster-123.9742	2.6075	4.1.1.49	pckA	Phosphoenolpyruvate carboxykinase (ATP)
Cluster-123.24230	2.6515	1.2.4.1	PDHA	Pyruvate dehydrogenase E1 component subunit alpha
Cluster-123.3335	3.9198		pckA	Phosphoenolpyruvate carboxykinase (ATP)
Cluster-123.20195	-2.5422	6.2.1.1	ACSS	Acetyl-coenzyme A synthetase, chloroplastic/glyoxysomal
Cluster-123.17385	4.8032	1.2.1.3	ALDH	Aldehyde dehydrogenase family 2 member C4
Cluster-123.8742	-2.4424	1.1.1.1	adhC	Alcohol dehydrogenase class-3
Cluster-123.11048	3.8678	2.3.3.1	CS	Citrate synthase 3
Cluster-123.14320	2.845	2.3.3.8	ACLY	ATP-citrate synthase alpha chain protein 2
Cluster-123.10240	-2.1295	1.1.1.49	G6PD	Glucose-6-phosphate 1-dehydrogenase, chloroplastic
Cluster-123.13628	2.076	1.1.1.37	MDH2	Malate dehydrogenase, chloroplastic
Pyruvate metabolism				
Cluster-123.10882	1.9071	4.2.1.2	fumC	Fumarate hydratase 1, mitochondrial
Cluster-123.16727	2.2582	2.5.1.18	GST	Probable glutathione S-transferase
Cluster-123.17049	3.3526		GST	Glutathione S-transferase F10
Cluster-123.10241	1.9626	1.1.1.44	PGD	6-phosphogluconate dehydrogenase, decarboxylating 1,
Cluster-123.17937	1.708		PGD	6-phosphogluconate dehydrogenase, decarboxylating 3,
Cluster-123.6804	2.719	1.11.19	speE,	Spermidine synthase 1
Pentose phosphate pathway				
Cluster-123.4365	5.7161	2.2.1.1	tktA	Transketolase-2, chloroplastic
Cluster-123.11061		2.2.1.2	talA	Transaldolase
Cluster-123.3117	2.2122	2.7.6.1	PRPS	Ribose-phosphate pyrophosphokinase 1
Starch and sucrose metabolism				
Cluster-123.13717	5.1777	3.2.1.26	INV	Beta-fructofuranosidase, insoluble isoenzyme 1
Cluster-123.17075	5.4602		INV	Beta-fructofuranosidase, insoluble isoenzyme
Cluster-123.27992	2.0396	3.2.1.39	GN1_2_3	Glucan endo-1,3-beta-glucosidase 3
Cluster-123.28520	5.9011		GN5_6	Glucan endo-1,3-beta-glucosidase 5
Cluster-123.28866	3.6581	2.4.1.1	PYG	Alpha-glucan phosphorylase, H isozyme
MVA pathway				
Cluster-123.21857	2.2947	2.3.3.9	atoB	Probable acetyl-CoA acetyltransferase, cytosolic 2
Cluster-123.15039	2.9083	2.3.3.10	HMGS	Hydroxymethylglutaryl-CoA synthase
Cluster-123.7404	2.6686	4.1.1.33	MVD	Diphosphomevalonate decarboxylase 2
Cluster-123.19817	2.4825	2.5.1.1	FDPS	Farnesyl pyrophosphate synthase
Cluster-123.19817	2.4825	2.5.1.10	FDPS	Farnesyl pyrophosphate synthase
Cluster-123.11329	3.0563	11414107	KAO	Ent-kaurenoic acid oxidase 1
Cluster-10761.0		1.14.11.12		Gibberellin 20 oxidase 1-B O
Cluster-123.14995	-3.0605	2.1.1.295		Tocopherol O-methyltransferase, chloroplastic
Cluster-123.16841		1.1.1.34	HMGCR	3-hydroxy-3-methylglutaryl-coenzyme A reductase 1
Cluster-24667.0			HMGCR	3-hydroxy-3-methylglutaryl coenzyme A reductase 2
Cluster-123.7834			HMGCR	3-hydroxy-3-methylglutaryl-coenzyme A reductase 3
MEP pathway				
Cluster-6682.0		5.3.32	IDI	Isopentenyl-diphosphate Delta-isomerase 1
Cluster-123.15944		2.2.1.7	dxs	Probable 1-deoxy-D-xylulose-5-phosphate synthase
Cluster-123.10600		1.1.1.267	dxr	1-deoxy-D-xylulose 5-phosphate reductoisomerase, chloroplastic
Cluster-123.14045		2.7.7.60	ispD	2-C-methyl-D-erythritol 4-phosphate cytidylyltransferase, chloroplastic
Cluster-123.25552		2.7.1.148	ispE	4-diphosphocytidyl-2-C-methyl-D-erythritol kinase
Terpenoid biosynthesis				
Cluster-123.16886	-1.6327	1.3.1.83	chlP	Geranylgeranyl diphosphate reductase, chloroplastic
Cluster-123.16886	-1.6327	1.1.3.83	chlP	Geranylgeranyl diphosphate reductase, chloroplastic

Cluster-123.16569	-2.4098		chlP	Geranylgeranyl diphosphate reductase, chloroplastic
Cluster-123.14998	-3.6757	2.1.1.95		Probable tocopherol O-methyltransferase, chloroplastic
Cluster-123.14995	-3.0605			Tocopherol O-methyltransferase, chloroplasti
		Plant hormone signal transduction		
Cluster-123.2178	4.3588	AUX1	AUX1, LAX	Auxin transporter-like protein 1
Cluster-123.7138	-1.7357	AUX1/IAA	IAA	Auxin-responsive protein IAA9
Cluster-25520.0	-4.5132		IAA	Auxin-responsive protein IAA13
Cluster-123.20707	-1.5695	GID1	GID1	Gibberellin receptor
Cluster-123.16771	7.0076	DELLA	DELLA	DELLA protein
Cluster-123.30722	6.0123	JAZR1	JAR1	Jasmonoyl--L-amino acid synthetase JAR6
Cluster-123.30386	6.6155		JAR1	Jasmonoyl--L-amino acid synthetase JAR6
Cluster-123.28001	8.4848	JAZ	JAZ	Protein TIFY 10A
Cluster-123.4168	8.1443	MYC2	MYC2	Transcription factor MYC2
Cluster-123.3941	3.9435		MYC2	Transcription factor MYC2

Supplementary Table S4. Regulatory network from the integrated analysis of miRNA-mRNA data

miRNA ID	Fold change	mRNA ID	Fold change	Description
dfr-miR156b	-8.5365	Cluster-10273.0	-1.7485	Squama promoter-binding-like protein 3
		Cluster-123.29223	3.5728	Arogenate dehydratase 3
		Cluster-123.29409	2.9169	Guanylate kinase 1
		Cluster-23659.0	-2.8911	L-type lectin-domain containing receptor kinase
		Cluster-123.13809	-2.8843	Pentatricopeptide repeat-containing protein
		Cluster-123.19695	-1.9519	CBS domain-containing protein CBSX3
		Cluster-123.8725	-1.6135	Putative glutathione peroxidase 7
		Cluster-123.5408	1.8182	Light-inducible protein CPRF2
		Cluster-123.1038	5.4348	Transcription factor MYB20
dfr-miR156c	-3.2	Cluster-10273.0	-1.7485	Squama promoter-binding-like protein 3
		Cluster-123.19313	1.7138	Probable serine/threonine-protein kinase SIS8
		Cluster-123.20543	-2.7227	Transcription repressor KAN1
		Cluster-123.6557	-1.6352	Pentatricopeptide repeat-containing protein
		Cluster-123.7903	1.7956	G protein-coupled receptor 7
		Cluster-123.16999	2.211	Probable protein phosphatase 2C
		Cluster-123.14057	2.6173	Histone H3.3-like type 2
		Cluster-123.25881	2.6414	Tuberculostearic acid methyltransferase
		Cluster-123.20114	2.7506	B2 protein
		Cluster-123.29223	3.5728	Arogenate dehydratase 3
		Cluster-123.28610	-2.091	Pentatricopeptide repeat-containing protein
		Cluster-123.23487	-1.6409	Molybdenum cofactor sulfurase
		Cluster-123.22583	2.0388	IAA-amino acid hydrolase ILR1-like 3
		Cluster-123.26549	-3.3717	Long-chain-alcohol oxidase
		Cluster-123.22415	-3.1846	Glucan endo-1,3-beta-glucidase 12
		Cluster-23659.0	-2.8911	L-type lectin-domain containing receptor kinase
		Cluster-123.18173	-2.621	Fructe-1,6-bisphphatase
		Cluster-123.17902	-1.9966	2-methylene-furan-3-one reductase
		Cluster-123.19695	-1.9519	CBS domain-containing protein CBSX3
		Cluster-123.20747	2.0049	Homeobox-DDT domain protein RLT1
		Cluster-123.22763	2.3964	Linamarin synthase 2
		Cluster-123.20103	2.6536	UDP-glycosyltransferase
		Cluster-123.24349	2.7405	O-fucosyltransferase 19
		Cluster-123.4022	2.9833	Transcription factor KUA1
		Cluster-123.23959	3.1297	Disease resistance protein TAO1
		Cluster-12165.0	3.3156	Histone H3.2
		Cluster-123.14806	3.4865	Scarecrow-like protein 14
dfr-miR160a	-8.0234	Cluster-123.3474	2.061	Auxin response factor 18
		Cluster-123.4068	2.054	Auxin response factor 16
		Cluster-123.2933	2.3179	Probable leucine-rich repeat receptor-like protein kinase
		Cluster-123.3200	2.5757	Beta-glucosidase 18
		Cluster-123.5888	5.5054	Mitochondrial carrier protein CoAc1
		Cluster-123.16555	-3.8488	Chalcone synthase
		Cluster-123.17914	-3.2321	photosystem I subunit PsaO
		Cluster-123.14287	-2.3081	RHOMBOID-like protein 9
		Cluster-123.20187	2.044	Membrane-anchored ubiquitin-fold protein 3
		Cluster-123.18740	2.306	Lysosomal beta glucosidase
		Cluster-123.4405	2.5092	Cytokinin hydroxylase

		Cluster-123.28431	2.8925	G-type lectin S-receptor-like serine/threonine-protein kinase
dfr-miR166a	-8.0234	Cluster-123.14967	2.2091	Disease resistance protein L6
		Cluster-123.10655	-2.4092	Calcium-transporting ATPase 8
		Cluster-123.16944	-2.9765	UDP-glycosyltransferase
		Cluster-123.9847	4.9293	Putative methyltransferase
dfr-miR171a	-19.5771	Cluster-123.10974	-2.3492	Scavenger receptor class B member 1
		Cluster-123.16151	-2.1084	Dormancy-associated protein 1
		Cluster-123.19430	2.969	Scarecrow-like protein 14
		Cluster-24393.0	-4.3188	Bifunctional nuclease 1
		Cluster-123.19430	2.969	Scarecrow-like protein 14
		Cluster-123.10411	3.3118	UDP-galactose/UDP-glucose transporter 7
		Cluster-123.27995	5.4762	Mitogen-activated protein kinase kinase kinase A
dfr-miR171b	0.2481	Cluster-123.19430	2.969	Scarecrow-like protein 14
		Cluster-123.10974	-2.3492	Scavenger receptor class B member 1
		Cluster-123.29383	2.1689	Zinc finger CCCH domain-containing protein 24
		Cluster-123.16739	-3.6975	Photosystem II core complex proteins psbY
		Cluster-123.16151	-2.1084	Dormancy-associated protein 1
		Cluster-123.11662	1.7213	Serine/threonine-protein kinase STY13
		Cluster-123.18601	2.2211	Serine/threonine-protein kinase PCRK2
		Cluster-26561.0	2.3745	Probable long-chain-alcohol O-fatty-acyltransferase 5
		Cluster-123.21047	3.8836	Unsaturated rhamnogalacturonyl hydrolase YteR
		Cluster-123.20658	5.4116	Probable L-type lectin-domain containing receptor kinase S.7
		Cluster-123.27185	-2.6199	Pentatricopeptide repeat-containing protein DOT4,
		Cluster-123.12783	2.0341	Probable serine/threonine-protein kinase PIX7
		Cluster-123.18228	2.0593	C2 and GRAM domain-containing protein At5g50170
		Cluster-123.23405	2.842	RHOMBOID-like protein 2
		Cluster-123.19430	2.969	Scarecrow-like protein 14
		Cluster-123.23589	3.5155	Probable sucrose-phosphate synthase 2
		Cluster-123.30629	4.6644	Calcium-dependent protein kinase 17
		Cluster-33680.0	4.694	Probable L-type lectin-domain containing receptor kinase
dfr-miR171c		Cluster-123.18266	2.255	Glutathione S-transferase F10
		Cluster-123.12337	3.2048	Endoplasmic reticulum oxidoreductin-1
		Cluster-123.18771	-5.8455	40S ribosomal protein S15
		Cluster-123.8398	-2.2953	Pentatricopeptide repeat-containing protein
		Cluster-123.9159	2.3147	FAS1 domain-containing protein
		Cluster-123.21840	2.5269	Aconitate hydratase
dfr-miR408	-2.72096	Cluster-123.10871	6.1936	Leucine-rich repeat receptor-like serine/threonine-protein kinase
		Cluster-123.14233	-2.0905	Ferredoxin-1
		Cluster-123.15627	1.622	Probable polyamine transporter
		Cluster-123.4444	3.7865	Light-sensor Protein kinase
		Cluster-22443.0	-2.8687	Leucine-rich repeat receptor-like serine/threonine-protein kinase
		Cluster-123.16299	1.6082	NADP-dependent glyceraldehyde-3-phphate dehydrogenase
